# Sleep Preserves Physiological Arousal in Emotional Memory

**DOI:** 10.1038/s41598-019-42478-2

**Published:** 2019-04-12

**Authors:** Jennifer E. Ashton, Marcus O. Harrington, Anna á Váli Guttesen, Anika K. Smith, Scott A. Cairney

**Affiliations:** 1 Department of Psychology, University of York, Heslington, York, YO10 5DD UK; 2 York Biomedical Research Institute (YBRI), University of York, Heslington, York, YO10 5DD UK

## Abstract

Traumatic experiences are associated with increased emotional arousal. Overnight consolidation strengthens the episodic content of emotional memories, but it is still unclear how sleep influences the associated arousal response. To investigate this question, we compared the effects of sleep and wake on psychophysiological and subjective reactivity during emotional memory retrieval. Participants provided affective ratings for negative and neutral images while heart rate deceleration (HRD) and skin conductance responses (SCRs) were monitored. Following a 12-hour delay of sleep or wakefulness, participants completed an image recognition task where HRD, SCRs and affective ratings were recorded again. HRD responses to previously-encoded (“old”) negative images were preserved after sleep but diminished after wakefulness. No between-group difference in HRD was observed for novel negative images at recognition, indicating that the effects of sleep for old images were not driven by a generalised overnight increase in visceral activity, or circadian factors. No significant effects of sleep were observed for SCRs or subjective ratings. Our data suggest that cardiac arousal experienced at the time of encoding is sensitive to plasticity-promoting processes during sleep in a similar manner to episodic aspects of emotional memory.

## Introduction

Distressing emotional experiences are associated with amplified levels of arousal, which constitutes the affective tone of resultant emotional memories^1^. Sleep facilitates memory consolidation; the process by which new and labile traces become strong and enduring representations^2^. Previous research has suggested that emotionally negative and neutral memories benefit from sleep^3–11^ (but see also^12^). Yet, while the impacts of sleep on the episodic details of emotional memories are well characterised, how overnight consolidation influences the affective tone of such memories – or *mnemonic arousal* – is unclear.

Two theoretical perspectives offer opposing predictions on the affective memory function of sleep. The first of these argues that sleep preserves both the content and the affective tone of emotional experiences, facilitating rapid decision making when faced with future threats or endangerment^13^. The alternative view proposes that the affective tone of emotional experiences becomes unbound from the episodic representation during overnight consolidation, weakening the associated arousal response when reflecting on negative past experiences^14,15^. To address these frameworks, several studies have compared changes in affective ratings for emotional images after post-encoding intervals of sleep and wakefulness. While some report a decrease in emotional reactivity across sleep (vs wake)^16,17^, others report a sleep-related preservation^13,18,19^ or intensification^20^ of mnemonic arousal.

These mixed results likely arise from a complex interplay between sleep and the dynamic inputs to subjective emotional appraisals, including physiological signals and behavioural goals^21^. To address this issue, other studies have incorporated psychophysiological measures into their paradigms^22^. Two such measures are heart rate deceleration (HRD) and the skin conductance response (SCR), which are mediated by parasympathetic and sympathetic mechanisms, respectively^23,24^. These autonomic indices map onto the affective tone of external stimuli. Hence, negative stimuli elicit greater HRD and SCRs than neutral stimuli^25–28^, permitting reliable, event-related assessments of affective arousal.

Employing HRD and SCRs, Pace-Schott and colleagues investigated the effects of a daytime nap, as compared to an equivalent period of wakefulness, on mnemonic arousal^25^. HRD responses to negative images were sustained across the nap but diminished across wakefulness, suggesting a sleep-related preservation of affective reactivity. However, the opposite pattern was observed for SCRs, which decreased across sleep and endured the wakeful interval. Taken together, these findings might imply that sleep exerts divergent effects on autonomic aspects of memory governed by sympathetic and parasympathetic processes. Subsequent research reported an overnight weakening of SCRs and HRD responses to object images, although these effects were observed for both negative and neutral stimuli, suggesting a generalised sleep-associated depotentiation of visceral activity^29^.

Recent work has investigated the effects of sleep on mnemonic arousal in children, employing HRD and affective ratings, as well as the EEG late positive potential (LPP; an index of arousal evoked by emotional stimuli)^21^. Compared to a day of wakefulness, sleep led to a decrease in emotional responses to negative images, as indicated by valence ratings and the LPP. In contrast, HRD reactivity to negative images was retained across sleep and weakened over wakefulness. It was suggested that sleep might preserve automatic emotional responses (i.e. HRD), but at the same time increase the accessibility of more complex emotional responses (i.e. subjective ratings and the LPP) to top-down cognitive control via a strengthening of affective cortical representations^21^.

Despite the advances arising from studies employing psychophysiological methods, only a limited number of experiments have attempted to differentiate between affective responses to previously-encoded images and those related to novel emotional stimuli^25^. It is therefore unclear whether the impacts of sleep on physiological reactivity reflect an overnight change in mnemonic arousal or a more generalised, sleep-related change in affect. We aimed to address this issue in the current study and gain clearer insights into the relationship between sleep and emotional memory processing.

Combining psychophysiological measures (HRD and SCRs) and subjective emotional ratings, we investigated the impacts of sleep and wakefulness on affective responses to previously-encoded and novel aversive images. Because prior work has suggested that sleep has divergent effects on parasympathetic and sympathetic components of mnemonic arousal^25^, we predicted that sleep would preserve HRD reactivity for previously-encoded negative images, but reduce the corresponding SCR. Based on convergent evidence that sleep increases top-down control of emotional responses at retrieval^21^, subjective ratings for previously-encoded negative images were predicted to become more positive after sleep. We further predicted that sleep would not impact on psychophysiological or subjective responses to novel images, indicating that the effects of sleep on emotional reactivity were specific to previously-encoded information.

## Results

**Figure 1:**
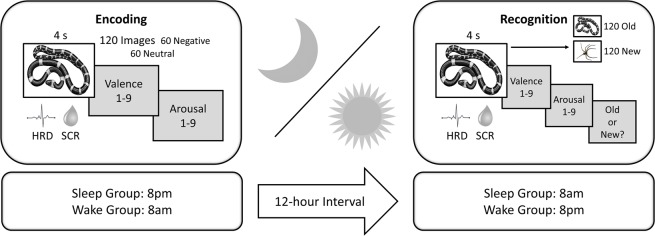
Experimental Procedures: At encoding, participants viewed emotionally negative and neutral images in the evening (sleep group) or morning (wake group). Participants returned 12 hours later for a recognition task in which they were re-exposed to the “old” images from encoding and saw an equal number of “new” images. In both sessions, heart rate deceleration (HRD) and skin conductance responses (SCRs) were recorded for each image, and participants provided 9-point ratings of valence and arousal. Participants also indicated at recognition whether they thought that each image was old or new. All images in this figure were obtained from Pixabay.com.

At encoding, participants rated the emotional valence (negative to positive) and arousal (boring to exciting) of negative and neutral images while HRD and SCRs were monitored (see Fig. 1). Following an interval of overnight sleep or daytime wakefulness, participants completed a recognition task in which the previously-encoded (“old”) images were interspersed with an equal number of unseen (“new”) images. Psychophysiological and subjective emotional responses were recorded again, and participants indicated whether they thought that each image was old or new. The overall recognition hit rate was near ceiling (see *Recognition Accuracy* below). As such, all old images were included in all subsequent analyses.

### Mnemonic Arousal

#### Heart Rate Deceleration

HRD was computed as the maximum beats-per-minute (BPM) deceleration during stimulus exposure relative to a 1 s baseline period that immediately preceded each trial^29^. HRD means for old images were applied to a 2 (Session: Encoding/Recognition) × 2 (Emotion: Negative/Neutral) × 2 (Group: Sleep/Wake) mixed ANOVA. A main effect of Emotion indicated that HRD reactivity was generally stronger for negative images than neutral images (*F*(1,46) = 34.50, *p* < 0.001, ƞ_p_^2^ = 0.43). There was no main effect of Session (*F*(1,46) = 1.75, *p* = 0.19, ƞ_p_^2^ = 0.04) indicating that, across both valences and groups, HRD did not differ between encoding and recognition. No main effect of Group indicated that HRD, collapsed across both sessions and valences, was comparable for the sleep and wake groups (*F*(1,46) = 1.04, *p* = 0.31, ƞ_p_^2^ = 0.02). There was no Emotion*Group interaction (*F*(1,46) = 0.05, *p* = 0.83, ƞ_p_^2^ = 0.001) indicating that, across both sessions, there were no between-group differences in HRD as a function of image valence. Between-session changes in HRD (across both valences) were greater for the wake group than the sleep group, but the Session*Group interaction was not significant (*F*(1,46) = 3.03, *p* = 0.09, ƞ_p_^2^ = 0.06). There was, however, a significant Session*Emotion interaction (*F*(1,46) = 9.57, *p* = 0.003, ƞ_p_^2^ = 0.17) indicating that between-session changes in HRD differed for negative and neutral images. Importantly, a significant Session*Emotion*Group interaction also emerged (*F*(1,46) = 4.07, *p* = 0.049, ƞ_p_^2^ = 0.08).

To clearly interpret this effect, we decomposed the Session*Emotion*Group interaction by the factor Emotion and performed mixed ANOVAs (factors: Session, Group) separately for negative and neutral images. For negative images, there was a main effect of Session (*F*(1,46) = 8.03, *p* = 0.007, ƞ_p_^2^ = 0.15) indicating that HRD responses were weaker at recognition than at encoding. Importantly, however, a Session*Group interaction indicated that this between-session decrease in HRD reactivity was only present in the wake group (*F*(1,46) = 7.19, *p* = 0.01, ƞ_p_^2^ = 0.14). No such effects were observed for neutral images (Session: *F*(1,46) = 0.08, *p* = 0.78, ƞ_p_^2^ = 0.002; Session*Group: *F*(1,46) = 0.29, *p* = 0.59, ƞ_p_^2^ = 0.006), and neither ANOVA revealed a main effect of Group (negative images: *F*(1,46) = 0.72, *p* = 0.40, ƞ_p_^2^ = 0.015; neutral images: *F*(1,46) = 1.26, *p* = 0.27, ƞ_p_^2^ = 0.027).

**Figure 2:**
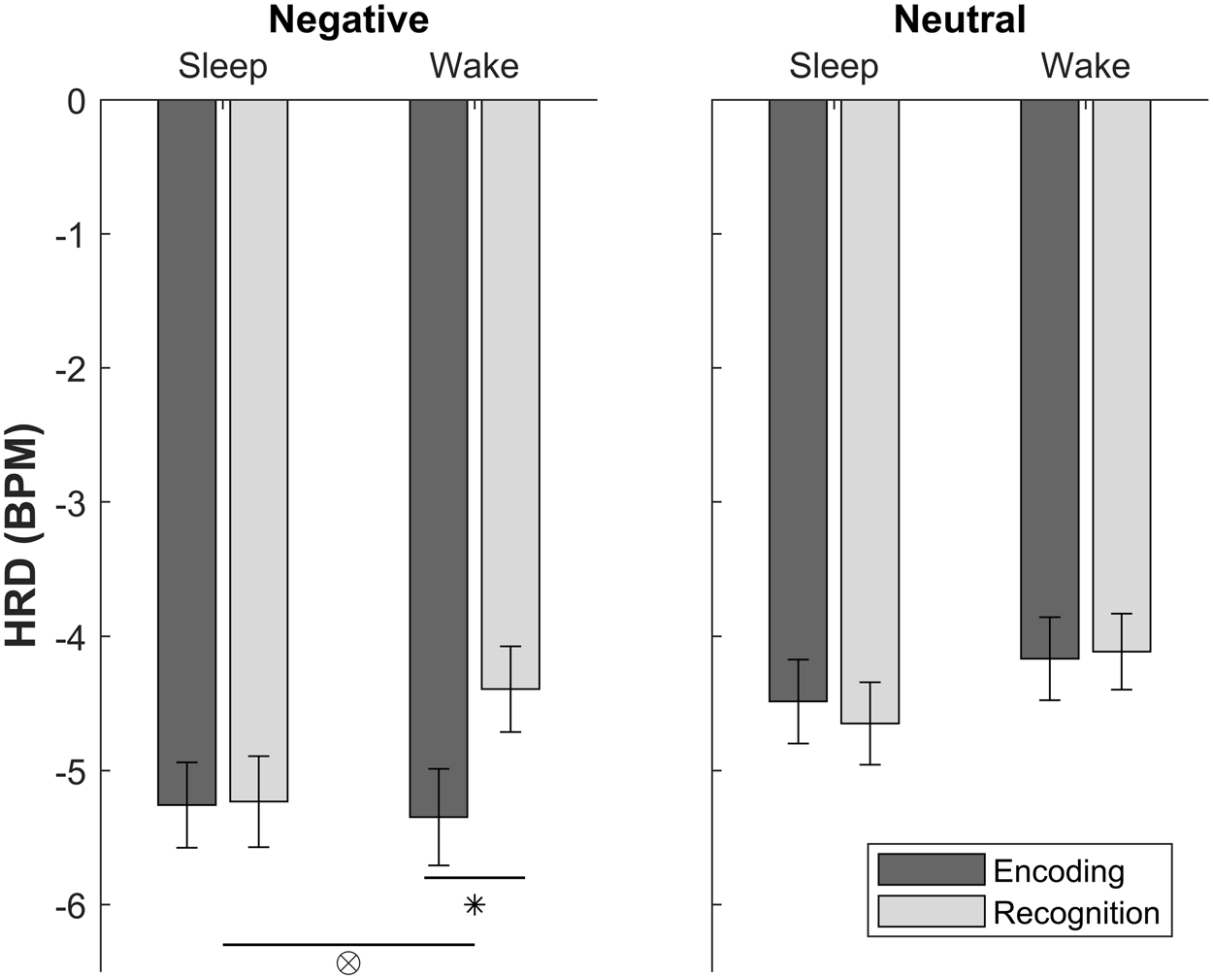
Heart Rate Deceleration (HRD): HRD responses at encoding and recognition for the sleep and wake groups presented separately for negative and neutral images. Significance markers are shown for the Session*Group interaction (⊗ *p* = 0.01) and for the negative and neutral image comparison (*p = 0.001). Data are shown as means (±SEM). BPM = beats per minute.

Taken together, these results indicate that HRD responses to negative images were preserved over sleep but diminished over wakefulness (see Fig. 2). This was confirmed by pairwise comparisons of HRD for negative images at encoding and recognition (wake group: *t*(23) = 3.62, *p* = 0.001; sleep group: *t*(23) = 0.12, *p* = 0.91). Summary statistics for the sleep group were applied to an additional Bayesian analysis using the JASP^©^ Summary Stats Module with a prior based on a Cauchy distribution and a scale of 0.707 (JASP default). The resulting Bayes factor (BF_01_ = 4.63) provided moderate support for the null effect (i.e. no between-session change in HRD for negative images in the sleep group), suggesting that cardiac arousal is retained over sleep. The sleep group’s self-reported sleep duration did not significantly correlate with overnight changes in HRD [recognition HRD-encoding HRD] for negative images (*r* = −0.19, *p* = 0.39).

To ensure that the effects of sleep were not driven by a generalised overnight increase in visceral activity, or circadian factors, we applied HRD means for new images to a 2 (Emotion: Negative/Neutral) × 2 (Group: Sleep/Wake) mixed ANOVA. HRD reactivity was again generally stronger for negative than neutral images (Emotion main effect: *F*(1,46) = 47.03, *p* < 0.001, ƞ_p_^2^ = 0.51). Importantly, however, there was no main effect of Group (*F*(1,46) = 1.87, *p* = 0.18, ƞ_p_^2^ = 0.04) and no Group*Emotion interaction (*F*(1,46) = 0.08, *p* = 0.78, ƞ_p_^2^ = 0.002). HRD data is presented in Table 1.

**Table 1:** Heart Rate Deceleration (HRD): HRD responses to negative and neutral images presented separately for the sleep and wake groups. New items refer to foil images that were presented at recognition alone. Data are shown as means (±SEM). BPM = beats per minute.

		HRD (BPM)		
		Encoding	Recognition	New
Sleep Group	Negative	−5.26 (±0.32)	−5.23 (±0.34)	−6.05 (±0.35)
	Neutral	−4.49 (±0.31)	−4.65 (±0.31)	−5.01 (±0.32)
Wake Group	Negative	−5.35 (±0.36)	−4.39 (±0.32)	−5.40 (±0.36)
	Neutral	−4.17 (±0.31)	−4.11 (±0.28)	−4.44 (±0.29)

In the sleep group, HRD reactivity for new images at recognition appeared to be increased relative to that observed at encoding. To investigate this observation further, we applied HRD means for novel images (i.e. those seen at encoding and new images seen at recognition) to a 2 (Session: Encoding/Recognition) × 2 (Emotion: Negative/Neutral) × 2 (Group: Sleep/Wake) mixed ANOVA. A main effect of Session (*F*(1,46) = 5.40, *p* = 0.025, ƞ_p_^2^ = 0.11) indicated that, across both groups and image valences, HRD reactivity to novel images increased between encoding and recognition. However, there was no main effect of Group and there were no significant interactions (*p* > 0.05). Unsurprisingly, a main effect of Emotion emerged, such that HRD reactivity was generally higher for negative than neutral novel images (*F*(1,46) = 56.79, *p* < 0.001, ƞ_p_^2^ = 0.55). Taken together with our main findings, this analysis suggests that the effects of sleep at recognition were specific to previously-encoded images.

#### Skin Conductance Responses

**Figure 3:**
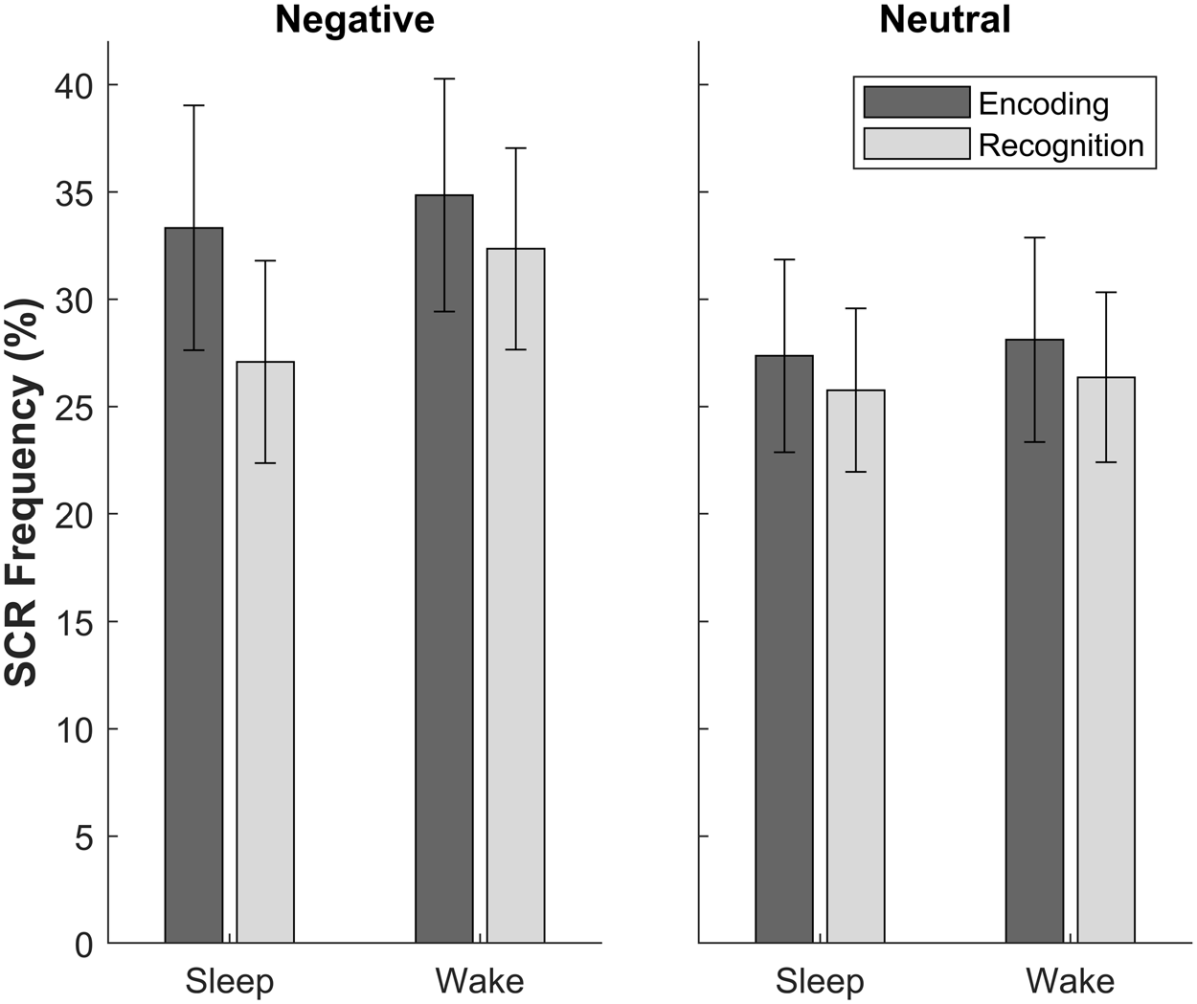
Skin Conductance Responses (SCR): SCR frequencies at encoding and recognition for the sleep and wake groups presented separately for negative and neutral images. Data are shown as means (±SEM).

Our analyses refer to SCR frequency, which is calculated within each condition as the percentage of trials that elicited a SCR^29^. Two wake group participants were non-responders (i.e. did not produce a single electrodermal response that met the threshold for an event-related SCR, see *Methods*) and were thus excluded from the SCR analyses. Square-root transformed SCR frequency means for old images were applied to a 2 (Session: Encoding/Recognition) × 2 (Emotion: Negative/Neutral) × 2 (Group: Sleep/Wake) mixed ANOVA. SCR frequencies were generally higher for negative than neutral images (Emotion main effect: *F*(1,44) = 10.20, *p* = 0.003, ƞ_p_^2^ = 0.19). There were no main effects of Session (*F*(1,44) = 1.26, *p* = 0.27, ƞ_p_^2^ = 0.03) or Group (*F*(1,44) = 0.16, *p* = 0.69, ƞ_p_^2^ = 0.004). The Session*Emotion*Group interaction did not emerge in this analysis (*F*(1,44) = 1.38, *p* = 0.25, ƞ_p_^2^ = 0.03), and no other significant interactions were present (*p* > 0.05, see Fig. 3).

For new images, SCR frequencies were again generally higher for negative than neutral items (Emotion main effect: *F*(1,44) = 5.67, *p* = 0.02, ƞ_p_^2^ = 0.11). There was no main effect of Group (*F*(1,44) = 0.12, *p* = 0.74, ƞ_p_^2^ = 0.003) and no Group*Emotion interaction (*F*(1,44) = 2.15, *p* = 0.15, ƞ_p_^2^ = 0.05). SCR data is presented in Table 2.

**Table 2:** Skin Conductance Responses (SCR): SCR frequencies for negative and neutral images presented separately for the sleep and wake groups. New items refer to foil images that were presented at recognition alone. Data are shown as means (±SEM).

		SCR Frequency (%)		
		Encoding	Recognition	New
Sleep Group	Negative	33.33 (±5.70)	27.08 (±4.72)	28.33 (±4.63)
	Neutral	27.36 (±4.49)	25.76 (±3.81)	25.35 (±3.53)
Wake Group	Negative	34.85 (±5.42)	32.35 (±4.70)	28.33 (±4.51)
	Neutral	28.11 (±4.77)	26.36 (±3.96)	20.98 (±2.89)

#### Subjective Emotional Ratings

Valence ratings for old images were applied to a 2 (Session: Encoding/Recognition) × 2 (Emotion: Negative/Neutral) × 2 (Group: Sleep/Wake) mixed ANOVA. Subjective valence ratings were generally lower for negative than neutral images (Emotion main effect: *F*(1,46) = 707.16, *p* < 0.001, ƞ_p_^2^ = 0.94). There were no main effects of Session (*F*(1,46) = 0.32, *p* = 0.57, ƞ_p_^2^ = 0.007) or Group (*F*(1,46) = 1.29, *p* = 0.26, ƞ_p_^2^ = 0.03). Between-session changes in valence ratings (across both negative and neutral images) were greater in the sleep group than the wake group, but the Session*Group interaction was not significant (*F*(1,46) = 3.40, *p* = 0.07, ƞ_p_^2^ = 0.07). There was a Session*Emotion interaction (*F*(1,46) = 5.41, *p* = 0.02, ƞ_p_^2^ = 0.11). Across both groups, valence ratings for negative images increased (i.e. became more positive) between encoding and recognition (*t*(47) = 2.18, *p* = 0.03), whereas valence ratings for neutral images decreased (i.e. became more negative, *t*(47) = 1.78, *p* = 0.08). These effects were not modulated by sleep (Session*Emotion*Group interaction: *F*(1,46) = 0.66, *p* = 0.42, ƞ_p_^2^ = 0.01) and the Emotion*Group interaction was not significant (*F*(1,46) = 0.48, *p* = 0.49, ƞ_p_^2^ = 0.01).

Arousal ratings for old images were applied to the same ANOVA. Subjective arousal ratings were generally higher for negative than neutral images (Emotion main effect: *F*(1,46) = 241.39, *p* < 0.001, ƞ_p_^2^ = 0.84). There were no main effects of Session (*F*(1,46) = 0.07, *p* = 0.79, ƞ_p_^2^ = 0.002) or Group (*F*(1,46) = 1.49, *p* = 0.23, ƞ_p_^2^ = 0.03), and no significant interactions (*p* > 0.05).

For new images, the same main effects of Emotion were found for valence ratings (*F*(1,46) = 591.09, *p* < 0.001, ƞ_p_^2^ = 0.93) and arousal ratings (*F*(1,46) = 206.14, *p* < 0.001, ƞ_p_^2^ = 0.82). There were no main effects of Group and no significant interactions (*p* > 0.05). Subjective rating data is presented in Table 3.

**Table 3:** Subjective Emotional Ratings: Arousal and valence ratings for negative and neutral images presented separately for the sleep and wake groups. New items refer to foil images that were presented at recognition alone. Data are shown as means (±SEM).

		Arousal Ratings			Valence Ratings		
		Encoding	Recognition	New	Encoding	Recognition	New
Sleep Group	Negative	6.59 (±0.12)	6.56 (±0.14)	6.70 (±0.14)	2.68 (±0.09)	2.77 (±0.11)	2.74 (±0.12)
	Neutral	4.56 (±0.14)	4.64 (±0.14)	4.75 (±0.15)	5.34 (±0.05)	5.34 (±0.08)	5.30 (±0.07)
Wake Group	Negative	6.70 (±0.13)	6.60 (±0.14)	6.79 (±0.15)	2.70 (±0.11)	2.77 (±0.11)	2.73 (±0.11)
	Neutral	4.21 (±0.16)	4.18 (±0.15)	4.29 (±0.15)	5.54 (±0.09)	5.42 (±0.08)	5.45 (±0.08)

### Recognition accuracy

Recognition accuracy was assessed with the sensitivity index (*d*’), which was calculated as: [Normalized (hits/(hits + misses))–Normalized (false alarms/(false alarms + correct rejections))]. We adopted a log-linear approach to safeguard this analysis against errors arising from 0 and 1 values: for each participant, 0.5 was added to the total hits and total false alarms, and 1 was added to the total signal (old) and total noise (new) trials^30^. Recognition accuracy scores (*d*’) were applied to a 2 (Emotion: Negative/Neutral) × 2 (Group: Sleep/Wake) mixed ANOVA. Negative images were generally better recognised than neutral images (Emotion main effect: *F*(1,46) 11.63, *p* = 0.001, ƞ_p_^2^ = 0.20). However, there was no main effect of Group (*F*(1,46) = 0.53, *p* = 0.47, ƞ_p_^2^ = 0.01) and no Emotion*Group interaction (*F*(1,46) = 2.69, *p* = 0.11, ƞ_p_^2^ = 0.06). Recognition accuracy data is presented in Table 4.

**Table 4:** Recognition Accuracy: Data refer to the percentage of old images scored as hits and misses, and the percentage of new images scored as correct rejections (CR) and false alarms (FA). Recognition accuracy was assessed via the sensitivity index (*d*’). Data are shown as means (±SEM).

		Hit	Miss	CR	FA	*d*’
Sleep Group	Negative	96.11 (±1.08)	3.89 (±1.08)	95.97 (±0.78)	4.03 (±0.78)	3.66 (±0.16)
	Neutral	94.93 (±1.59)	5.07 (±1.59)	95.49 (±0.78)	4.51 (±0.78)	3.50 (±0.13)
Wake Group	Negative	96.89 (±0.64)	3.11 (±0.64)	96.21 (±0.59)	3.79 (±0.59)	3.67 (±0.12)
	Neutral	92.34 (±1.77)	7.65 (±1.77)	95.23 (±0.70)	4.77 (±0.70)	3.26 (±0.13)

## Discussion

We combined psychophysiological and subjective measures to investigate the effects of sleep on mnemonic arousal (i.e. the affective tone of emotional memories). HRD responses to previously-encoded negative images were preserved across sleep but diminished over wakefulness. No between-group difference in HRD was observed for novel images at recognition, indicating that the effects of sleep for old images were not due to a generalised overnight increase in cardiac arousal, or time-of-day effects. Between-session changes in SCRs to negative images, by contrast, were comparable after sleep and wakefulness. Negative images received more positive subjective evaluations across the delay, whereas neutral images became more negatively evaluated. However, these effects were not modulated by sleep.

The observed effects of sleep on cardiac arousal are reminiscent of those reported in previous work. Pace-Schott *et al*. found that HRD responses to negative images were sustained across a post-encoding nap but diminished across a corresponding period of wakefulness^25^. Unlike the current study, however, Pace-Schott *et al*. also observed a sleep-related decrease in SCRs to negative images. Interestingly, participants in Pace-Schott *et al*. were repeatedly exposed to a small number of images at encoding, with SCRs showing strong intra-session habituation. This early electrodermal habituation might have been consolidated during the subsequent nap period, facilitating a further between-session reduction in SCR reactivity. Given that participants in the current study received only a single exposure to the negative stimuli at encoding, baseline SCR habituation may have been insufficient to foster any overnight change. It should also be noted that our criteria for defining event-related SCRs (see *Methods*) meant that several trials were excluded from the analyses. Reduced statistical power (as compared to the HRD data) may have therefore compromised our ability to detect a sleep-related modification in electrodermal arousal. Future studies that assess SCRs on more trials (e.g. using SCR amplitudes) might provide greater sensitivity to such overnight changes.

How is cardiac mnemonic arousal preserved during overnight sleep? HRD responses to external stimuli are mediated by the parasympathetic nervous system^24^. Autonomic control of cardiac activity shifts towards parasympathetic dominance with the onset of non-rapid eye movement (NREM) sleep, and this parasympathetic supremacy continues throughout the NREM period^31,32^. Given that NREM sleep constitutes the majority of overnight sleep, the related increase in parasympathetic control might bolster HRD reactivity to aversive images, which otherwise habituates across a corresponding period of wakefulness. Yet, by this account, one might expect an increase in HRD responses for all negative stimuli after sleep, irrespective of whether encoding has already taken place. Since the effects of sleep on HRD reactivity in the current study were only observed for previously-encoded images, it is possible that the cardiac arousal associated with emotional experiences is sensitive to overnight consolidation. Further research using polysomnography can address this possibility by investigating the components of sleep linked to the preservation of cardiac mnemonic arousal in emotional memory.

A prominent *Active Systems* model of sleep-associated plasticity posits that overnight consolidation occurs via a process of neural reactivation in learning-related brain networks^2,33,34^. Recent work has suggested that highly salient representations, such as those pertaining to personally-relevant or emotionally-charged experiences, are preferentially reactivated and thereby strengthened in the sleeping brain^35–38^. Since memory reactivation in sleep is thought to predominate during NREM periods (when parasympathetic influences on cardiac activity are maximal)^2,34^, a tantalising albeit speculative possibility is that mnemonic replay supports the stabilisation of memory units pertaining to both the content of emotional experiences (as observed previously^37,39^) and the associated cardiac arousal. Consistent with this idea, recent work has suggested that consolidation during sleep strengthens multiple memory traces corresponding to the same encoding event^40^. Future research might test this possibility via the targeted delivery of memory cues during NREM sleep, which typically improves retention^37,39,41–43^. If offline memory replay stabilises cardiac mnemonic arousal, memory cueing in NREM sleep might result in more durable HRD responses to previously-encoded negative stimuli.

In recent work, Cunningham *et al*. observed an overnight decrease in HRD reactivity to *both* negative and neutral stimuli^29^. However, participants in Cunningham *et al*. made recognition judgements on separate object and background components of scenes, which were extracted from the images originally presented at encoding. It is possible that presenting objects in the absence of associated background contexts weakened effective discrimination of aversive from non-aversive stimuli, preventing a selective, sleep-related retention of cardiac arousal from emerging during negative image recognition. This is, nevertheless, a speculative proposal that requires further investigation.

Using a similar paradigm to the current study but in children, Bolinger *et al*. observed a sleep-related preservation of amplified HRD responses to negative (vs neutral) images^21^. Yet, sleep was additionally found to promote a generalised decrease in HRD reactivity to all images. Such nuanced effects of sleep on cardiac mnemonic arousal in adults and children might be related to the marked changes in sleep architecture that are known to accompany development^44^.

Negative images were rated more positively after the delay, whereas neutral images were rated more negatively. However, between-session changes in subjective valence ratings were unaffected by sleep. Previous studies addressing the impacts of sleep on self-reported emotional reactivity have produced mixed results, with evidence for reduced^16,17^, preserved^13,18,19^ or increased^20^ subjective affect. Subjective emotional ratings require the integration of multiple inputs, including physiological signals and behavioural goals. Dynamic changes in the ratio of such inputs at encoding might influence the subsequent impacts of sleep, resulting in divergent behavioural outcomes. Interestingly, the changes in valence ratings observed in the current study were also reported in recent work with children, but only after sleep^21^.

Although recognition accuracy was not our primary outcome measure, we note that there was no significant difference in memory performance between the sleep and wake groups. Given the known benefits of sleep for memory consolidation^2,33,34^, this finding might appear surprising, particularly as several studies have reported a sleep-related improvement in emotional image recognition^6,7^. However, overall hit rates in the current study were near ceiling, potentially masking a sleep-associated memory gain.

In summary, our data indicate that HRD responses to negative images are preserved across overnight sleep. Supported by an increase in parasympathetic activity, cardiac mnemonic arousal might be sensitive to plasticity-promoting processes during NREM sleep in a similar manner to the episodic components of emotional memory. This sleep-associated preservation of cardiac arousal may represent an adaptive mechanism to guide behaviour when faced with the same future ordeal. It should be noted, however, that excessive and protracted retention of mnemonic arousal could pose negative consequences for health and wellbeing.

## Methods

### Participants

Sixty-one healthy, right-handed adults were randomly assigned to a sleep group (n = 34) or a wake group (n = 27). Participants were compensated with £25 or BSc Psychology course credit. Thirteen participants were excluded for the following reasons: excessive movement/noise in the psychophysiological data (8), failure to follow task instructions (1), PC-network malfunction (3) and experimenter error (1). We report on the data from the remaining 48 participants (sleep group: n = 24, 7 male, mean ± SD age = 20.04 ± 1.77 years; wake group: n = 24, 9 male, age = 19.96 ± 1.60). Participants had no history of sleep, psychiatric or cardiac disorders, were medication free, had not consumed alcohol or caffeine during the preceding 24 hours and were non-smokers. Written informed consent was obtained from all participants in line with the Research Ethics Committee of the Department of Psychology at the University of York, who approved the study. All study methods were carried out in accordance with the British Psychological Society’s Code of Ethics and Conduct.

### Stimuli

Four-hundred and eighty pictures were taken from the International Affective Picture System (IAPS)^45^ and the Nencki Affective Picture System (NAPS)^46^. IAPS and NAPS images range from everyday scenes to images of injury, violence and contaminated foods. The images were divided into two sets of 240 (A and B). Participants were only ever exposed to set A or B (counterbalanced across participants in both groups), thus ensuring that any effects of sleep on mnemonic arousal were generalised across different sets of images. Each set contained 120 negative images and 120 neutral images. For the assigned set, half of the images (60 negative and 60 neutral) were presented at both encoding and recognition, while the other half were presented as foils at recognition alone (see *Procedure*).

Each image in the IAPS/NAPS databases is accompanied with a 9-point rating of valence (i.e. 1 = highly negative, 5 = neutral, 9 = highly positive) and arousal (i.e. 1 = non-arousing, 9 = highly arousing). To ensure that the stimuli used in this study were balanced, we applied the valence and arousal ratings for each image to a 2 (Image Set: A/B) × 2 (Phase: Encoding/Recognition) × 2 (Emotion: Negative/Neutral) ANOVA. For valence ratings, negative images (mean ± SEM, 2.62 ± 0.04) were significantly lower than neutral images (5.56 ± 0.04, *F*(1,479) = 2382.48, *p* < 0.001). There were no other significant main effects or interactions (*p* > 0.05). For arousal ratings, negative images (mean ± SEM, 6.67 ± 0.04) were significantly higher than neutral images (4.28 ± 0.04, *F*(1,479) = 1671.88, *p* < 0.001). No other significant main effects or interactions were observed (*p* > 0.05). In the current study, participant ratings of the images were consistent with our assignment of stimuli to the negative and neutral categories (see *Results*).

### Procedure

The study was divided into two sessions: (1) encoding and (2) recognition (see Fig. 1). The sleep group began the encoding phase at ~8 pm and returned for the recognition phase at ~8 am the following morning. Participants in the sleep group each provided subjective estimations of the number of hours slept between sessions (mean ± SD = 8.25 ± 0.98). The wake group completed the encoding and recognition phases in the morning and evening, respectively.

Participants completed the Epworth Sleepiness Scale (ESS)^47^ and Beck Anxiety Inventory (BAI)^48^ at the beginning of the encoding session. Before both sessions, participants completed the Stanford Sleepiness Scale (SSS)^49^. There were no group differences in ESS scores (median, sleep group = 4, wake group = 5, U = 227.00, *p* = 0.20) or BAI scores (mean ± SEM, sleep group = 6.67 ± 1.26, wake group = 5.00 ± 1.04, *t*(46) = 1.02, *p* = 0.31). There were no group differences in SSS scores at encoding (median; sleep group = 2, wake group = 2, U = 268.00, *p* = 0.62) or recognition (median; sleep group = 2, wake group = 2.5, U = 213.00, *p* = 0.10). Between-session changes in self-reported sleepiness did differ between groups, however (median; sleep group = 0, wake group = 1, U = 175.00, *p* = 0.01). Nonparametric Mann-Whitney U tests were employed due to violations of normality.

A short (~2 min) electrocardiography (ECG) recording was obtained from each participant immediately before encoding and recognition. Resting heart rate was derived from the first 1 min of ECG data following a 30 s acclimatisation period. There were no group differences in resting heart rate (beats per minute, BPM) at encoding (median; sleep group = 71.86, wake group = 72.90, U = 253.00, *p* = 0.47) or recognition (median; sleep group = 70.08, wake group = 74.63, U = 263.00, *p* = 0.61). Between-session changes in resting heart rate did not differ between groups (median; sleep group = 2.52, wake group = 0.20, U = 274.00, *p* = 0.77).

### Encoding

Each trial began with a 500 ms fixation period. A randomly selected image was then presented for 4 s. Participants were instructed to focus on the image for the entire time that it was presented. For the purposes of collecting SCRs, the image offset was followed by a 4 s delay. Participants then provided ratings of emotional valence and then arousal using the Self-Assessment Manikins (SAMs)^50^ without time limit. The SAMs consist of two series of images depicting nine levels of emotional valence (1 = highly negative or unpleasant, 5 = neutral, 9 = highly positive or pleasant) and arousal (1 = non-arousing or boring; 9 = highly arousing or exciting). A varied delay of 3.5 s, 4.5 s, 5.5 s or 6.5 s (counterbalanced and randomised across trials) then followed before the next trial. This jittered inter-stimulus interval helped to ensure reliable SCR detection^51^ while, at the same time, minimising the duration of each experimental session. Each image was presented once. At the end of the task, participants were instructed to go home and sleep as normal (sleep group) or to go about their normal daily routine and not nap (wake group) before returning ~12 hours later for the recognition session.

### Recognition

The recognition task included the “old” images seen at encoding plus a randomly interspersed set of unseen “new” images (see *Stimuli*). After 500 ms of fixation, a randomly selected image was presented for 4 s. Following an additional 4 s delay, participants rated the valence and arousal of the image using the same 9-point SAMs as at encoding. Participants then indicated whether they thought that the image was old (i.e. they recognised the image from encoding) or new (i.e. they had not seen the image before). To ensure that participants were reasonably confident in their recognition judgements, they were also asked to provide a certainty rating for their old/new response using a 4-point scale (1 = absolutely sure, 2 = fairly sure, 3 = not very sure, 4 = not sure at all). A varied delay of 3.5 s, 4.5 s, 5.5 s or 6.5 s (counterbalanced and randomised across trials) preceded the next trial.

The certainty ratings indicated that participants were highly confident in their recognition judgements. Across all participants, the mean ± SEM certainty rating for hits (i.e. correct “old” responses) was 1.13 ± 0.01. Only 11 out of 48 participants provided a certainty rating of 4 (i.e. “not sure at all”) for one or more of the correctly recognised images. Across all participants, only 0.38% of correctly recognised trials were accompanied with a certainty rating of 4. Given that participants were highly confident in their memory judgements, we included all recognition trials in our analyses. Certainty rating data and analyses are available in the Supplementary Information.

### Equipment

#### Experimental Tasks

Behavioural tasks were presented with E-Prime version 2.0 (Psychology Software Tools, Inc.) and projected onto a 56″ screen placed ~2 m from where participants were seated. Behavioural responses were made on a keyboard using the dominant (right) hand.

#### HRD and SCR

Psychophysiological measures were recorded using a BIOPAC MP36R data acquisition system and AcqKnowledge (ACQ) 4.4.1 software. The E-Prime-triggered square pulse outputs were transmitted to the MP36R unit via a BIOPAC STP35A interface enabling precise alignment of each stimulus onset to the ECG and SCR data.

For HRD, two BIOPAC EL503 ECG electrodes were attached to the midline of the right clavicle and the lower left rib. ECG data were sampled at 2 kHz. ACQ software derived interbeat intervals from the ECG series to calculate heart rate in BPM. Heart rate was estimated for 5 one-second bins starting 1 s prior to stimulus onset and continuing for a further 4 s. The first 1 s bin acted as a pre-stimulus baseline. HRD was computed by subtracting the heart rate baseline value from the minimum heart rate value during the next four 1-s bins^29^. Accordingly, HRD refers to the maximum BPM deceleration during the 4 s of stimulus exposure.

For SCR, two BIOPAC EL507 disposable adhesive electrodes were attached to the fingertips of the index and middle fingers of the non-dominant (left) hand. Tonic electrodermal activity (EDA) was sampled at 2 kHz and then downsampled to 62.5 Hz for analysis. A median smoothing filter with a window of 63 samples was used to remove artifacts by eliminating transient spikes from the electrodermal signal^52^. A 1 Hz FIR low-pass filter was then applied to attenuate noise in the data. Phasic EDA waveforms were constructed by applying ACQ’s smoothing baseline removal method to the tonic EDA waveforms (baseline estimation window width =5 s). An event-related SCR was identified by the ACQ detection algorithm as an increase in electrical conductance ≥0.01 µS within a window of 1 to 6 s post stimulus onset. SCR frequency was calculated within each condition as the percentage of trials that met the threshold for an event-related SCR^29^. The SCR frequency data were square-root transformed to correct for non-normal distributions^53^.

## Supplementary information

Supplementary Information

## Data Availability

Study data can be retrieved via the following link: https://osf.io/wbfdu/?view_only=fdb92038f9d1458cb73b94871ad99bee.
