# Supplementary material for: Sleep Preserves Physiological Arousal in Emotional Memory
Source: Sci Rep. 2019 Apr 12;9:5966. doi: 10.1038/s41598-019-42478-2 (PMC6461689; doi:10.1038/s41598-019-42478-2)
Supplement: Supplementary file 1 — Supplementary Information [file 41598_2019_42478_MOESM1_ESM.docx]

**SUPPLEMENTARY INFORMATION**

Sleep Preserves Physiological Arousal in Emotional Memory

Jennifer E. Ashton^a^, Marcus O. Harrington^a^, Anna á Váli Guttesen^a^, Anika K. Smith^a^, Scott A. Cairney^a, b^**^*^**

1. Department of Psychology, University of York, Heslington, York, YO10 5DD, UK
2. York Biomedical Research Institute (YBRI), University of York, Heslington, York, YO10 5DD, UK

**Valence Ratings**

There were three outliers in the sleep group at encoding and/or recognition (outlier threshold = ±3 SDs from group mean). Presented below are the results when these outliers were excluded.

*Old Images: 2 (Session: Encoding/Recognition) x 2 (Emotion: Negative/Neutral) x 2 (Group: Sleep/Wake) mixed ANOVA:*

| Effect | *F* | *p* | ƞ_p_^2^ |
| --- | --- | --- | --- |
| Session | 0.02 | .89 | <.001 |
| Emotion | 859.61 | <.001* | .95 |
| Group | 2.42 | .13 | .05 |
| Session*Emotion | 10.35 | .002* | .19 |
| Session*Group | 1.89 | .18 | .04 |
| Emotion*Group | 0.97 | .33 | .02 |
| Session*Emotion*Group | 0.01 | .92 | <.001 |

*New Images: 2 (Emotion: Negative/Neutral) x 2 (Group: Sleep/Wake) mixed ANOVA:*

| Effect | *F* | *P* | ƞ_p_^2^ |
| --- | --- | --- | --- |
| Emotion | 719.03 | <.001* | .94 |
| Group | 2.04 | .16 | .05 |
| Emotion*Group | 1.56 | .22 | .04 |

**Arousal Ratings**

There were two outliers (sleep group n=1, wake group n=1) at encoding and/or recognition (outlier threshold = ±3 SDs from group mean). Presented below are the results when these outliers were excluded.

*Old Images: 2 (Session: Encoding/Recognition) x 2 (Emotion: Negative/Neutral) x 2 (Group: Sleep/Wake) mixed ANOVA:*

| Effect | *F* | *p* | ƞ_p_^2^ |
| --- | --- | --- | --- |
| Session | 0.01 | .91 | <.001 |
| Emotion | 257.37 | <.001* | .85 |
| Group | 3.67 | .06 | .08 |
| Session*Emotion | 1.62 | .21 | .04 |
| Session*Group | 0.24 | .63 | .005 |
| Emotion*Group | 3.39 | .07 | .07 |
| Session*Emotion*Group | 0.06 | .80 | .001 |

*New Images: 2 (Emotion: Negative/Neutral) x 2 (Group: Sleep/Wake) mixed ANOVA:*

| Effect | *F* | *p* | ƞ_p_^2^ |
| --- | --- | --- | --- |
| Emotion | 244.55 | <.001* | .85 |
| Group | 2.30 | .14 | .05 |
| Emotion*Group | 4.28 | .05* | .09 |

The Emotion*Group interaction was not found in the analysis of new images reported in the main text. Decomposing this interaction, participants in the sleep group rated new neutral images as more arousing than participants in the wake group (*t*(44)=3.36, *p*=.002), whereas there was no between-group difference in arousal ratings for new negative images (*t*(44)=0.44, *p*=.66).

**Certainty Ratings**

Participants provided a certainty rating for each recognition response (1=absolutely sure, 2=fairly sure, 3=not very sure, 4=not sure at all). Mean (± SEM) certainty ratings for hits (i.e. correct “old” responses) are presented below.

|  | **Negative Images** | **Neutral Images** |
| --- | --- | --- |
| **Sleep Group** | 1.11 (± 0.02) | 1.18 (± 0.04) |
| **Wake Group** | 1.09 (± 0.02) | 1.16 (± 0.04) |

Certainty ratings for hits were applied to a 2 (Emotion: Negative/Neutral) x 2 (Group: Sleep/Wake) mixed ANOVA. There was a main effect of Emotion *F*(1,46)=15.49, *p*<.001, ƞ_p_^2^=.25), indicating that participants were more certain in their correct recognition responses for negative images than neutral images. There was no main effect of Group (*F*(1,46)=0.42, *p*=.52, ƞ_p_^2^=.009) and no Emotion*Group interaction (*F*(1,46)=0.005, *p*=.94, ƞ_p_^2^<.001).

**Correlations between Measures**

Exploratory correlations between HRD, SCRs, valence ratings and arousal ratings to negative images at encoding (across all participants) were conducted. The correlation between valence and arousal ratings was significant (*r*=-.63, *p*<.001) indicating that negative valence ratings were associated with higher arousal ratings. Interestingly, the correlation between HRD and valence ratings was also significant (*r*=.36, *p*= .01), indicating that positive valence ratings were associated with diminished HRD responses. No other correlation was significant (*p*>.05).
